# Engagement With a Health Information Technology–Augmented Self-Management Support Program in a Population With Limited English Proficiency: Observational Study

**DOI:** 10.2196/24520

**Published:** 2021-05-11

**Authors:** Leah Machen, Margaret A Handley, Neil Powe, Delphine Tuot

**Affiliations:** 1 University of California, San Francisco San Francisco, CA United States

**Keywords:** automated telehealth intervention, limited English proficiency, mHealth, language problems, telehealth, SMS, text message

## Abstract

**Background:**

Limited English proficiency (LEP) is an important driver of health disparities. Many successful patient-level interventions to prevent chronic disease progression and complications have used automated telephone self-management support, which relies on patient activation and communication to achieve improved health outcomes. It is not clear whether these interventions are similarly applicable to patients with LEP compared to patients with English proficiency.

**Objective:**

The objectives of this study were as follows: (1) To examine the impact of LEP on patient engagement (primary outcome) with a 12-month language-concordant self-management program that included automated telephone self-management support, designed for patients with chronic kidney disease (CKD). (2) To assess the impact of LEP on change in systolic blood pressure (SBP) and albuminuria (secondary outcomes) resulting from the self-management program.

**Methods:**

This was a secondary analysis of the Kidney Awareness Registry and Education (KARE) pilot trial (NCT01530958) which was funded by the National Institutes of Health in August 2011, approved by the University of California Institutional Review Board in October 2011 (No. 11-07399), and executed between 2013 and 2015. Multivariable logistic and linear models were used to examine various facets of patient engagement with the CKD self-management support program by LEP status. Patient engagement was defined by patient’s use of educational materials, completion of a health coaching action plan, and degree of participation with automated telephone self-management support. Changes in SBP and albuminuria at 12 months by LEP status were determined using multivariable linear mixed models.

**Results:**

Of 137 study participants, 53 (38.7%) reported LEP, of which 45 (85%) were Spanish speaking and 8 (15%) Cantonese speaking. While patients with LEP and English proficiency similarly used the program’s educational materials (85% [17/20] vs 88% [30/34], *P*=.69) and completed an action plan (81% [22/27] vs 74% [35/47], *P*=.49), those with LEP engaged more with the automated telephone self-management support component. Average call completion was 66% among patients with LEP compared with 57% among those with English proficiency; patients with LEP requested more health coach telephone calls (*P*=.08) and had a significantly longer average automated call duration (3.3 [SD 1.4] min vs 2.2 [1.1 min], *P*<.001), indicating higher patient engagement. Patients with LEP randomized to self-management support had a larger, though nonstatistically significant (*P*=.74), change in SBP (–4.5 mmHg; 95% CI –9.4 to 0.3) and albuminuria (–72.4 mg/dL; 95% CI –208.9 to 64.1) compared with patients with English proficiency randomized to self-management support (–2.1 mmHg; 95% CI –8.6 to 4.3 and –11.1 mg/dL; 95% CI –166.9 to 144.7).

**Conclusions:**

Patients with LEP with CKD were equally or more engaged with a language-concordant, culturally appropriate telehealth intervention compared with their English-speaking counterparts. Augmented telehealth may be useful in mitigating communication barriers among patients with LEP.

**Trial Registration:**

ClinicalTrials.gov NCT01530958; https://clinicaltrials.gov/ct2/show/NCT01530958

## Introduction

In 2019 the US Census Bureau reported that there were over 25 million people living in the United States classified as having limited English proficiency (LEP), defined by not answering “very well” regarding their ability to speak English [1]. Not only is LEP linked to lower socioeconomic status and poverty, but it is also a known predictor of poorer health status, decreased preventive care, and less access to medical care [2,3]. The obstacles to receiving medical care for these patients are further compounded by communication barriers, facilitating poor engagement with health care providers, worse interpersonal care and patient satisfaction, and less patient education [4-6]. Because of these factors, the LEP population may be a growing marginalized group of patients with high disease burden and poor connections with the health care system [1].

Automated telephone self-management support systems are a form of health information technology (HIT) that use patient vignettes to deliver education and self-management tools to patients and interactive voice response with an option to request clinician phone calls to engage patients in their health care. Automated telephone self-management support systems have been shown to improve clinical outcomes such as systolic blood pressure (SBP), depressive symptoms, and obesity, and reduce hospital admissions and mortality for patients with heart failure [6-10]. They have also been shown to have high levels of patient engagement, even among older adults who may be unable to use other forms of HIT due to limited vision, literacy, or technology knowledge [11]. This dynamic and interactive tool offers a unique opportunity to reach marginalized populations with poor health access and promote engagement in populations such as those with LEP.

Despite long-time awareness of the existence of this population and the gaps in their care, providers are only recently beginning to develop targeted health interventions to improve access and outcomes for patients with LEP. Patient engagement, encompassing patient activation which is defined as having the motivation, knowledge, skills, and confidence to make effective decisions to manage one’s health, has been shown to be associated with improved patient lifestyle choices, adherence, and chronic disease management [12-18]. Thus, finding novel ways to augment patient engagement will serve as one of many essential pathways to improve health care access and outcomes among the LEP population.

Automated telephone self-management support systems represent a group of novel interventions that can be tailored to specific patient populations. We sought to explore whether the LEP status impacted patient engagement with a language-concordant self-management program that featured automated telephone self-management support systems among participants with chronic kidney disease (CKD) randomized to the intervention arm of the Kidney Awareness Registry and Education (KARE) pilot trial. We hypothesized that patients with LEP would have higher levels of engagement than those with English proficiency given the novelty of a language-concordant intervention in an environment with a paucity of non-English self-management support materials.

## Methods

### Study Design

We conducted a retrospective analysis of the KARE pilot trial to assess participant engagement with and impact of an HIT-augmented comprehensive self-management support program on change in SBP and albuminuria among patients with limited (vs adequate) English proficiency. Details of the KARE study have been previously described [19]. In brief, KARE was a 2 × 2 factorial pilot randomized controlled trial that took place in 2 primary care clinics in San Francisco’s public health care delivery system. The study was funded by the National Institutes of Health in August 2011 and approved by the University of California Institutional Review Board in October 2011 (No. 11-07399). A total of 137 patients were enrolled in the trial, which was executed between 2013 and 2015. Overall results of the pilot trial have been published previously [20].

The KARE study had 2 levels of randomization. First, within each clinic, primary care practice teams consisting of several physicians (including trainees), 1 nurse, nurse practitioners, medical assistants, and behaviorists, were randomized 1:1 to 1 of 2 arms with a random number generator: access to a CKD registry versus usual care registry. Second, within 6 months of the provider-level randomization, eligible patients were recruited to a baseline visit and were randomized within each provider 1:1 to participate in a year-long comprehensive CKD self-management program.

Eligible patients included adults (≥18 years) with CKD, defined by 2 values of estimated glomerular filtration rate of 15-60 mL/min/1.73 m^2^ or albuminuria (urine dipstick ≥1+ or urine albumin-to-creatinine ratio >30 mg/g) documented in the electronic health record on 2 occasions at least 90 days apart, who had contact with their primary health care team at least once within the past 2 years and spoke English, Spanish, or Cantonese. Patients who spoke Spanish or Cantonese were monolingual and did not speak English. Patients were excluded from the study if they were recipients for kidney transplantation, pregnant, or were unlikely to benefit from the self-management support program due to hearing or visual impairment, impaired cognition or severe mental illness, or a life expectancy less than 6 months.

### Intervention

The KARE interventions have been previously described in detail and found to be acceptable among providers and patients [19,21]. In brief, the provider intervention consisted of an electronic health record–enabled CKD registry tool with “in-reach” and “outreach” elements to support team-based management of CKD. The patient intervention was a comprehensive CKD self-management support program with 3 distinct elements. The first element consisted of language-concordant, low-literacy written patient educational materials [22] couriered to patients at months 1, 4, and 8. The second element was a language-concordant and culturally tailored interactive automated telephone self-management program with 26 modules delivered every other week that provided education and self-management strategies related to topics pertinent to kidney health: basics of kidney disease and its association with hypertension; importance of participation in healthy behaviors (diet, physical activity, smoking cessation, stress reduction); avoidance of nonsteroidal anti-inflammatory medications; participation and preparation for clinic visits; complementary medication use; medication adherence; and glycemic control. In addition to providing educational content, the automated phone calls included “quiz questions” which patients would answer with a touch-tone phone throughout the module. These questions promoted engagement with the program and allowed patients to hear additional content or request telephone calls from their health coach. The third element of the CKD self-management support program consisted of telephone-based health coaching delivered by lay bilingual health coaches trained in motivational interviewing and action planning who called patients upon their request via the interactive telephone program and on an as-needed basis. Multimedia Appendix 1 includes an example English script for 1 week’s content related to use of nonsteroidal anti-inflammatory medications, as well as the corresponding health coach guide to promote the development of an action plan related to this topic.

### Study Outcomes

The primary outcome of this secondary analysis was patient engagement with the intervention determined a priori by the following measures which have been previously defined as engagement metrics for similar interventions [23]: percentage of users who engaged with any aspect of the automated system (defined by responding to at least one automated telephone self-management support call), percentage of automated calls completed, percentage of users who completed at least 80% of calls, and percentage of patients who created at least one action plan. We also a priori identified other engagement metrics that we believed fit the CKD self-management support program, including self-reported use of educational materials during study visits, duration of participation in the automated telephone calls determined by the telephone software (longer duration suggesting greater engagement due to interactive answering of health questions, repeating patient vignettes, and call completion), and number of requests for health coach callbacks from study participants during their automated telephone call (greater number of requests suggesting greater engagement).

Secondary outcomes of this study included changes in SBP and albuminuria severity (spot urinary albumin-to-creatinine ratio) from baseline to 12 months, both ascertained at study visits. SBP was measured with a digital blood pressure monitor (model HEM-907X; Omron), using the average of 3 SBP measurements in the right arm after the patient sat quietly for 5 min.

### Covariates

At the baseline visit, patient sociodemographic data (age, gender, race/ethnicity, education, income, insurance status) were self-reported, as were comorbidity data (diabetes, coronary artery disease, hyperlipidemia). Food insecurity and health literacy were ascertained using validated screening questionnaires [24].

### Statistical Analysis

We examined baseline characteristics of KARE participants and tested for differences by LEP status using chi-square tests for categorical variables and nonparametric Kruskal–Wallis tests for continuous variables. Multivariable logistic and linear regression models were used to determine differences in engagement with the self-management support program by LEP status adjusted for age, sex, and variables that differed by LEP status (race/ethnicity, education, insurance status). Impact of the self-management support program on change in SBP at 12 months and change in albuminuria by LEP status were determined using linear mixed models accounting for clustering by provider and controlling for sociodemographic variables and baseline measures of SBP and albuminuria, respectively.

## Results

### Participant Demographics

Of the 137 KARE participants, roughly half were male (66/137, 48.2%) with a mean age of 55.3 (SD 12.2) years; 8 of 137 participants were White (5.8%), 59 Black (43.1%), 49 Hispanic (35.8%), and 20 Asian (14.6%); 1 (0.7%) participant declined to answer. Over one-third (53/137, 38.7%) of participants reported LEP with primary languages of Spanish (45/53, 85%) and Cantonese (8/53, 15%); 71 out of 137 participants were high-school educated (51.8%) and 47/137 college (34.3%) educated, with lower numbers in the LEP group, 27/53 (51%) and 7/53 (13%), respectively, compared with the English-speaking group (*P*<.001 for both). In the overall cohort, 34/137 (24.8%) participants were uninsured or covered by a health care access program to subsidize medical care for uninsured residents of San Francisco, with 21/53 (40%) participants being in the LEP group and 13/84 (15%) in the English-speaking group (*P*<.001). Over three-quarters (91/121, 75.2%) of participants who provided these data reported annual income less than US $15,000 and 72 participants (52.6%) reported food insecurity, without any differences by LEP status. All study participants had CKD, of which 38.7% (53/137) had hypertension and 51.8% (71/137) had diabetes (Table 1).

**Table 1:** Sociodemographic characteristics of the study patients.

Characteristics			Overall (n=137)		Non-LEP (n=84)		LEP (n=53)		*P* value
Age (years), mean (SD)			55.3 (12.2)		56.3 (10.9)		53.7 (13.9)		.22
**Sex, n (%)**									.11
	Male	66 (48.2)		45 (53.6)		21 (39.62)			
	Female	71 (51.8)		39 (61.31)		32 (60.4)			
**Race/Ethnicity, n (%)**									<.001
	White	8 (5.8)		8 (9.6)		0 (0.0)			
	Black	59 (43.1)		59 (71.1)		0 (0.0)			
	Hispanic	49 (35.8)		4 (4.8)		45 (84.9)			
	Asian	20 (14.6)		12 (14.5)		8 (15.1)			
**Language, n (%)**									<.001
	English	84 (61.3)		84 (100.0)		0 (0.0)			
	Spanish	45 (32.8)		0 (0.0)		45 (84.9)			
	Cantonese	8 (5.8)		0 (0.0)		8 (15.1)			
**Education, n (%)**									<.001
	Primary school	19 (13.9)		0 (0.0)		19 (35.9)			
	High school/Technical education	71 (51.8)		44 (52.4)		27 (50.1)			
	College	47 (34.3)		40 (47.6)		7 (13.2)			
**Insurance, n (%)**									<.001
	None or HSF	34 (24.8)		13 (15.5)		21 (39.6)			
	Medicaid	61 (44.5)		44 (52.4)		17 (32.1)			
	Medicare	37 (27.0)		26 (30.9)		11 (20.8)			
	Other	5 (3.6)		1(1.2)		4 (7.6)			
**Income^a^, n (%)**									.43
	<15K	91 (75.2)		56 (77.8)		35 (71.4)			
	15-50K	30 (24.8)		16 (22.2)		14 (28.5)			
Food insecurity			72 (52.6)		42 (58.3)		30 (56.6)		.45
Health literate			101 (73.7)		63 (75.0)		38 (71.7)		.67
Hypertension (≥140/90 mmHg)			53 (38.7)		37 (69.8)		16 (30.2)		.11
Diabetes			71 (51.8)		39 (54.9)		32 (45.1)		.11
**CKD stage, n (%)**									.23
	CKD stages 1 and 2	46 (33.6)		25 (29.8)		21 (39.6)			
	CKD Stages 3 and 4	91 (66.4)		59 (70.2)		32 (60.4)			

^a^n=137 for all rows except income, for which n=121 (n=72 for non-LEP and n=49 for LEP).

### Primary Outcomes

**Figure 1:**
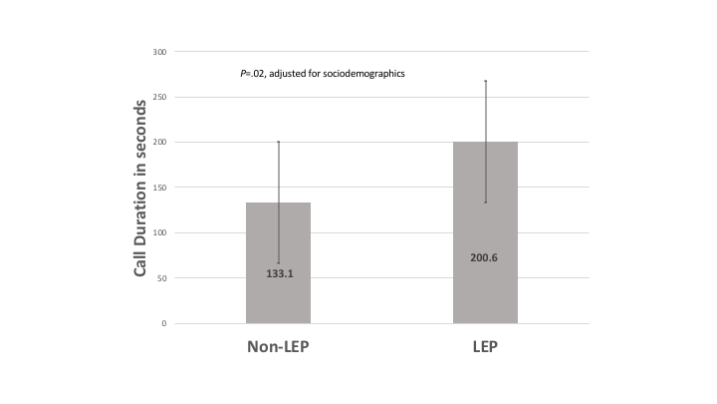
Mean (SD) of automated telephone self-management call duration by English proficiency. LEP: limited English proficiency.

Participant engagement measures pertinent to all 3 components of the CKD self-management support program included self-reported use of written education materials, development of at least one action plan, and degree of interaction with the automated telephone self-management support program. The overall use of patient education materials was 87% (47/54) and 57/74 (77%) participants developed an action plan. There were similar rates of self-reported use of educational materials among participants in the non-LEP and LEP group: 88% (30/34) and 85% (17/20), respectively (*P=*.73). Similarly, 35/47 (74%) participants in the non-LEP group and 22/27 (81%) participants in the LEP group developed at least one health-oriented action plan (*P=*.49). By contrast, engagement with the automated telephone self-management support differed by LEP status. While nearly all participants completed at least one automated telephone self-management support module (only 1 patient with English proficiency did not complete any automated telephone self-management support modules), the average completion rate of all modules was 57% among those with English proficiency and 66% among those with LEP. In multivariable linear regression, LEP status was positively associated with a higher automated telephone self-management support module completion rate (coefficient=0.3/10% higher completion), although this was not statistically significant (*P*=.75). “High engagers” were classified as participants who completed at least 80% of automated phone calls, of which 61% (17/28) were in the non-LEP group and 39% (11/28) were in the LEP group; however, there was a nonstatistically significant difference in this regard (*P=*.70). Among participants who did complete the automated telephone self-management support calls, the mean number of health coach callbacks requested was significantly larger in the LEP group compared with the non-LEP group (16 [SD 14.8] vs 11 [SD 10.6]; *P*=.004); however, in the adjusted model this outcome did not reach statistical significance (*P=*.08). The average call duration among the LEP group was 200.6 seconds compared with 133.1 seconds in the non-LEP group, a difference which was statistically significant when adjusted for age, sex, race/ethnicity, education, and insurance status (*P=*.02; Figure 1)

### Secondary Outcomes

**Figure 2:**
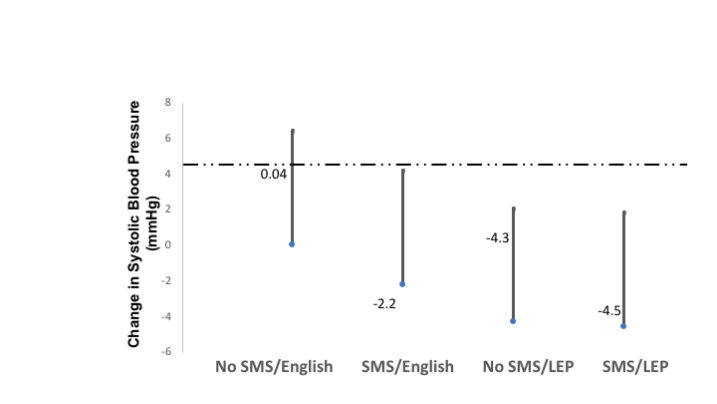
Estimated change in systolic blood pressure (95% CI) by intervention and English proficiency. LEP: limited English proficiency.

Change in SBP was nonstatistically greater in the LEP group compared with the non-LEP group among patients randomized to the self-management support intervention as well as among those randomized to usual care (*P=*.74). Adjusted estimates of SBP change among patients with LEP who received the self-management support intervention were –4.5 mmHg (SD 2.49) and among patients with LEP randomized to usual care were –4.3 mmHg (SD 2.97). Participants with English proficiency randomized to the intervention had an average adjusted estimated SBP change of –2.2 mmHg (SD 3.29), whereas those randomized to usual care did not experience any change in SBP (0.04; SD 3.26; Figure 2). Similarly, patients with LEP randomized to the self-management support intervention had a nonstatistically significant greater decrease in urine albumin-to-creatinine ratio compared with their English-speaking counterparts (–72.4 mg/dL [95% CI –208.9 to 64.1] vs –11.1 mg/dL [95% –166.9 to 144.7], *P*=.29).

## Discussion

### Principal Findings

In this study of primary care patients with CKD who participated in a trial examining the impact of a comprehensive CKD self-management support program, we found that participants with LEP showed higher engagement than those with English proficiency with the HIT elements of the program by completing the automated telehealth modules more often, spending significantly longer time with each automated module, and requesting more health coach phone callbacks for further information/clarification. The LEP status did not seem to influence engagement with the more traditional health education components of the program such as language-concordant reading materials and developing at least one action plan with a health coach. These data suggest that language-concordant HIT may offer a unique opportunity to impact the health of linguistically marginalized patients. However, despite differences in engagement, the comprehensive CKD self-management support intervention had a similar null impact on change in SBP and albuminuria among both patient groups (ie, those with English proficiency and those with LEP with kidney disease).

### Patient Engagement

Patient engagement has been shown to be associated with preventative behaviors such as participating in health screenings, regularly attending physician appointments, and improving dietary choices and exercise habits [12-18]. More highly engaged patients have also been shown to be less likely to smoke or consume illicit substances [25]. Further, among patients with chronic disease, higher engagement has been correlated with increased home monitoring, better treatment adherence, and more consistent medical follow-up [14,16,26-31]. Chronic diseases such as hypertension, diabetes, and kidney disease not only dominate in prevalence compared with acute diseases in the American population, but they also require consistent medical management, significant disease education, and higher levels of treatment adherence. Thus, the emphasis on patient engagement, activation, and education is paramount in our population.

### Impact of Limited English Proficiency

In general, LEP is associated with poorer health status, decreased access to medical care, reduced preventive health services, and poorer quality of care [2,32-36]. Patients with LEP are more likely to have poorly controlled hypertension and worse glycemic control among those with diabetes as outpatients, as well as higher infection rates and longer hospitalizations as inpatients [37-39]. In addition, patients with LEP are more likely to have lower socioeconomic status, compounding health disparities by adding the burden of poverty, limited education, and unemployment [3]. These disparities are only partially mitigated by patient-provider language concordance or interpreter use, as LEP has been shown to be an independent predictor of poorly controlled disease [32,40,41]. With over 25 million Americans having LEP, the impact of these disparities has prompted research into innovative programs to improve health status, preventive services, and patient activation within this population [1].

### Health Information Technology

Automated telephone self-management support has been highlighted by the Department of Health and Human Services as a potential tool to ameliorate health care for LEP populations, and has also been studied as an intervention in high utilizer and chronic disease cohorts [42,43]. Prior studies have shown that interventions with automated telephone self-management support can reduce hospitalizations and emergency department visits, assess medication adherence, monitor patient safety events, and improve outcomes such as medication and appointment adherence, immunizations and screening, and patient safety triggers [44,45]. However, while some studies suggest improvements in clinical outcomes such as diabetes control, the literature is lacking on studies determining efficacy of automated telephone self-management support on management of many chronic diseases [44,46,47]. While many prior trials included patients from diverse backgrounds, subanalyses of differences between patients with English proficiency and LEP were always not conducted. To our knowledge, this is one of few studies to analyze the impact of an intervention including automated telephone self-management support between patients with LEP and those with English proficiency.

### Conclusions

HIT is a burgeoning field with smartphone apps, telephone interventions, and interactive electronic medical record systems rapidly modifying the delivery of health care. The results of this study indicate that language-concordant automated telephone self-management support can be used equally well by patients with LEP compared with patients with English proficiency, with potentially even higher levels of engagement. This suggests that that language-concordant telehealth interventions could be useful in mitigating communication barriers known to negatively impact health status. This is of particular importance now, given the increase in novel telehealth modalities for care delivery in the era of COVID-19, with the number of telehealth visits more than doubling in some health systems in 2020 [48]. This surge in HIT use has also emphasized the “digital divide” where marginalized populations are less likely to have access to computers or internet services, and thus unable to participate in virtual clinic visits [49]. Because of these limitations and time needed to develop infrastructure to facilitate virtual visits, telephone encounters have become a mainstay of patient care [50].

Our findings also indicate that patients with LEP may more readily engage with language-concordant telehealth interventions as opposed to language-concordant reading materials. Reasons for this difference are likely multifactorial, but may include the fact that reading materials have to account for language, literacy, and numeracy, whereas telehealth interventions (often phone or video based) can convey similar ideas with clinical vignettes and stories, without the potential pitfalls associated with low literacy, numeracy, or even limited technological knowledge (which may contribute to lack of patient portal or web-based interventions) [23]. Thus, HIT customized to account for linguistic variation should be a key consideration in the development of novel telehealth tools.

The main limitation of this study is its small sample size, limiting power to examine the impact of the self-management support program on clinical outcome by LEP status. In addition, it was conducted in a single public health care system in California. As such, the results may not be generalizable to other settings or patient populations. However, we included a diverse patient population with a high percentage of patients with LEP.

HIT interventions are rapidly evolving to address patient needs, improve health outcomes, and increase patient engagement. As these innovations are used to increasingly engage our patients, and to the extent that the LEP population continues to grow in the United States, it is important that we develop them in a way to include, not ignore, the needs of this marginalized population. Language-concordant versions of automated telehealth and HIT tools have the potential to help bridge the disparities gap for the LEP population, and represent a unique opportunity to improve health outcomes in a disproportionately disease-burdened population.

## Appendix

Multimedia Appendix 1 English script for 1 week’s content.

## Abbreviations

CKD: chronic kidney disease
IT: information technology
KARE: Kidney Awareness Registry and Education
LEP: limited English proficiency
SBP: systolic blood pressure
