# Supplementary material for: Engagement With a Health Information Technology–Augmented Self-Management Support Program in a Population With Limited English Proficiency: Observational Study
Source: JMIR Mhealth Uhealth. 2021 May 11;9(5):e24520. doi: 10.2196/24520 (PMC8205419; doi:10.2196/24520)
Supplement: Multimedia Appendix 1 [file mhealth_v9i5e24520_app1.docx]

| ATSM item | Questions | Automated Education (depending on patient response; used to guide education during callback) | Guide scripts for KARE health coach telephone calls |
| --- | --- | --- | --- |
| W4Q1 | “During the past 7 days, how many days have you taken any of the following prescription or over-the-counter pain relievers: Ibuprofen, Advil, Motrin, Aleve, Excedrin?  Press the number of days.” | *That’s great. Did you know that certain pain pills, such as Ibuprofen, Advil, Motrin, Aleve may cause kidney injury? Remember that Aspirin for your heart is ok. Please talk to your doctor about whether these medications are safe for you.”*  *“Sounds like you have had some pain this past week. Do you take these medications for arthritis pain?*  *You might be going through something similar to Mrs. Jones. Mrs. Jones has arthritis in her knees and used to tak Aleve every day to treat the pain and keep up with her grandchildren. When she saw her doctor for a check-up, he told her that her kidneys were not working well. He told her that certain pain pills, such as Ibuprofen, Advil, Aleve, could cause some kidney injury. Mrs. Jones now ices her knee every afternoon for 10 minutes before she meets up with her grandchildren and no longer needs Aleve everyday.”* | Nonjudgmental: “It’s important for the doctors to understand how their patients are taking all their prescription and over-the-counter medications.”  Check accuracy: “In this week’s call, you answered that take these pain pills for arthritis pain and would like to avoid these types of pain pills if possible. Is that correct?  Check understanding about pain pills:   - “What over-the-counter pills are you currently taking now? For each one, tell me their names, how much you take, and what they are for?” - Do you have the bottles? Can you get them and read the name / instructions on them?” - “Tell me more about your pain and what you have tried to treat it.” - NSAIDs may be a risk factor for progressive CKD. It is associated with an acute decline in GFR and kidney function. - “What have you noticed about your health condition since you started taking the pills?” (Possible side effects include stomach pain, nausea, heart burn, increased BP and risks for diabetes and elderly patients)   Assess prior care:   - “Does your doctor know about this?” - “Is your doctor or someone else (like social worker or pharmacist) helping you with the pain?”   Community resources:   - Local swimming pools (Balboa, Sava, Coffman, Garfield, Hamilton, MLK Jr, Mission Community, North Beach, Rossi)   Help patient problem solve:   - Provide education about medications and reasons to take. - *Problem:* must walk for exercise; can’t go in pool - Solution(s): non-weight bearing activities: biking or water aerobics. - *Problem:* only Aleve works. - Solution(s): try Tylenol, heat/cold packs (can use dish towels that you run under scalding or really cold water); talk to doctor about getting a joint injection if it’s a knee/wrist/elbow/shoulder - *Problem*: only Advil works for headaches; Tylenol doesn’t work - Solution: talk to doctor; maybe a migraine medicine could help, or maybe find ways that help avoid the headaches altogether; sometimes headaches come from taking too many pain pills (sounds counter-intuitive, but true!) - Help patient set an action plan, using triggers or cues for remembering. (What small steps would you like to take to get started? What, when, how much, how often, how confident are you?) - Talk to your doctor and see what to do to avoid taking NSAIDs for a long period of time and understand if any risk factors apply to you - Alternatives for NSAIDs: meditation, behavioral modification, relaxation techniques, light exercise, deep breathing, music therapy   Follow-up:   - “Talk with your doctor more about this. He/she wants to know and to help you find a way to put you on the best possible treatment plan”   “I will check back to see how you are doing.” |

| ATSM item | Questions | Automated Education (depending on patient response; used to guide education during callback) | KARE telephone scripts for health coaches |
| --- | --- | --- | --- |
| W4Q7 | If it’s not for arthritis, do you take pills for a chronic injury?  Do you want to talk to a member of your health care team about ways to avoid these types of pain pills? | *“Sounds like you might be similar to Mr. Lee. Mr. Lee has had back pain every day since an accident 5 years ago. He thought the only way to make it through the day was to take 2 Motrin pills. Mr. Lee also has kidney disease from diabetes. One day, his doctor told him his kidney function was worse than usual. He advised Mr. Lee to stop taking Motrin and showed him some exercises he could do to strengthen his back and decrease the pain. The exercises didn’t work right away, so he took Tylenol initially But after doing the exercises every other day for a few weeks, Mr. Lee no longer needed pain pills as frequently. Not only did his back feel better, but his kidney function returned back to its previous level because he stopped taking Motrin every day.”* | Check accuracy: “In this week’s call, you answered that you wanted to talk with someone about your pain pills. Is that correct?”  Ask why requested call back: “Tell me what you’d like to talk about.” “Tell me why you are taking the pain pill.”  Address issues raised by patient:  Ex: side effects of taking the OTC pain pills  Provide education/resources: (from American College of Rheumatology)  - NSAIDs may be a risk factor for progressive CKD. It is associated with an acute decline in GFR and kidney function.  - Nonsteroidal anti-inflammatory drugs are commonly used as pain medicines in adults.  - NSAIDs are used to relieve pain and reduce signs of inflammation such as fever, swelling and redness.  - NSAIDs can decrease inflammation, such as in arthritis.  - Though NSAIDs can be obtained over the counter, you should always talk to your doctor before taking any.  - Do not take an over-the counter NDSAID if you are taking a prescription NSAID  *Use the coaching guide from previous ATSM triggers on NSAID Avoidance)  Follow-up:   - IF SERIOUS SYMPTOMS (dizziness, low sugar, chest pain, breathing problems) 🡪 REFER FOR URGENT OR EMERGENT EVALUATION - If questions about medications, notify clinic and encourage patient to follow-up in near future. |
